# Comparing the risk of anaphylaxis requiring epinephrine in oral immunotherapy and subcutaneous immunotherapy: A review of recent Canadian real-world literature

**DOI:** 10.1016/j.jacig.2023.100080

**Published:** 2023-02-08

**Authors:** Uliana Kovaltchouk, Samira Jeimy, Lianne Soller, Kara Robertson, Elissa M. Abrams, Scott B. Cameron, Harold Kim, Edmond S. Chan

**Affiliations:** a Division of Clinical Immunology and Allergy, Department of Medicine, Western University, London, Ontario, Canada; b Department of Pediatrics, Division of Allergy and Immunology, University of British Columbia, Vancouver, British Columbia, Canada; c Department of Pediatrics, Section of Allergy and Clinical Immunology, University of Manitoba, Winnipeg, Manitoba, Canada; d Division of Clinical Immunology and Allergy, Department of Medicine, McMaster University, Hamilton, Ontario, Canada

**Keywords:** Peanut oral immunotherapy, subcutaneous immunotherapy, systemic reactions

## Abstract

**Background:**

The safety of pediatric food oral immunotherapy (Ped-OIT) has been depicted by some as less favorable than subcutaneous immunotherapy (SCIT) owing to the increased number of serious adverse events requiring epinephrine. A review of real-world data comparing Ped-OIT and SCIT safety is necessary to guide shared decision making.

**Objectives:**

Our aim was to compare the safety and adverse event profiles of peanut Ped-OIT and SCIT using Canadian real-word literature.

**Methods:**

We performed a retrospective review of recent Canadian real-world literature on peanut Ped-OIT and SCIT safety and adverse events.

**Results:**

The incidences of systemic reactions requiring epinephrine were 11 in 270 patients (4.07%) and 12 in 41,020 doses (0.029%) in a multicenter study in British Columbia, Alberta, Manitoba, and Nova Scotia studying 270 preschool-age children treated with peanut OIT. Similarly, a multicenter study in South-Western Ontario examining 160 patients between the ages of 1 and 17 years who were treated with peanut OIT showed that the incidences of systemic reactions requiring epinephrine were 5 in 160 patients (3.1%) and 8 in 52,751 doses (0.015%). A single-center retrospective review of 380 patients receiving aeroallergen SCIT showed that the incidences of systemic reactions requiring epinephrine were 28 in 380 patients (7.4%) and 1 in 1047 injection visits (0.095%). These findings are comparable to those of a review of 860 patients in Ontario who received either aeroallergen or venom SCIT, in which the incidence of systemic reaction requiring epinephrine was 10 in 4242 injections (0.24%).

**Conclusion:**

Despite differences in the OIT protocols used and age groups studied, recent real-world data suggest that the safety of preschool peanut OIT or peanut OIT using a slower buildup schedule is comparable to that of SCIT.

Allergen immunotherapy has been established as an effective therapy for managing allergic rhinitis, allergic conjunctivitis, allergic asthma, stinging insect hypersensitivity, and food hypersensitivity.^1^ Subcutaneous immunotherapy (SCIT) involves subcutaneous administration of allergens in increasing doses until an effective dose in inducing clinical tolerance is reached.^1^ The most common adverse event associated with aeroallergen and venom SCIT entails local reactions; however, systemic reactions are the most serious adverse event. The rate of systemic reactions is low, and several randomized controlled studies, meta-analyses, and recent case series support the safety of SCIT.^2^, ^3^, ^4^, ^5^, ^6^ The combination of safety and efficacy has resulted in SCIT becoming a common treatment modality offered in both academic and community allergy practices. Like SCIT, oral immunotherapy (OIT) is an allergen-specific approach, but it involves introduction of an allergenic food into the diet in increasing doses. The aim of the therapy is to desensitize patients to specific food allergen(s) to decrease the risk of future allergic reactions and anaphylaxis. The safety of pediatric OIT (Ped-OIT) has been depicted by some as less favorable than SCIT owing to the increased number of serious adverse events requiring epinephrine.^7^ Although these conclusions have in part previously resulted in a proportion of allergy practices in certain countries not offering Ped-OIT to patients,^8^^,^^9^ Canadian allergy practices have been more open to including OIT in their treatment approach for food allergy.^10^ Importantly, the recently published Canadian clinical practice guidelines for Ped-OIT favorably recommended the use of Ped-OIT outside the research setting.^11^ Other than Ped-OIT, no alternative therapeutic options for patients with food allergies are recommended for use outside research in the Canadian guidelines. Because of this substantial unmet need for patients, there continues to be an urgent need to review and compare real-word data on the safety of Ped-OIT with that of other immunotherapy modalities.

## Results

Although Ped-OIT has historically been considered less safe, emerging evidence demonstrates similar safety profiles between Ped-OIT and SCIT. Recent Canadian real-world literature shows that the results of 2 peanut Ped-OIT studies are consistent with respect to safety and adverse events. A multicenter study collaborating between community and academic allergists in British Columbia, Alberta, Manitoba, and Nova Scotia studied 270 preschool-age children treated with peanut OIT between 2017 and 2018.^12^ Over a period of 16 to 22 weeks, patients received buildup dosing of peanut protein biweekly.^12^ Of the 270 patients, 243 reached maintenance dosing of 300 mg of peanut protein and 27 patients dropped out during the buildup.^12^ Although 67.8% of patients experienced a reaction during buildup, the majority of these reactions were either grade 1 or 2 (36.3% and 31.1%, respectively),^12^ as determined by using a modified World Allergy Organization Subcutaneous Immunotherapy Reaction Grading system.^12^ The overall incidences of systemic reactions requiring epinephrine were 11 in 270 patients (4.07%) and 12 in 41,020 peanut OIT doses (0.029%), with rates of epinephrine administration of 0.26% of clinic-administered doses, and 0.016% of doses administered at home.^12^ Similarly, a multicenter study collaborating between community and academic allergists in South-Western Ontario studied 160 patients between the ages of 1 and 17 years who were treated with peanut Ped-OIT between 2016 and 2020.^13^ Over a period of 14 months, the patients received monthly incremental increases in peanut protein.^13^ Of the 160 patients, 129 completed peanut Ped-OIT, reaching a maintenance dose of 250 mg of peanut protein, and 31 patients (19%) discontinued peanut Ped-OIT because of side effects.^13^ The incidences of systemic reactions requiring epinephrine were 5 in 160 patients (3.1%) and 8 in 52,751 peanut Ped-OIT doses (0.015%); whether epinephrine was administered at home or at the clinic was not reported.^13^ No patient mortalities were reported in either of these studies.

**Fig 1:**
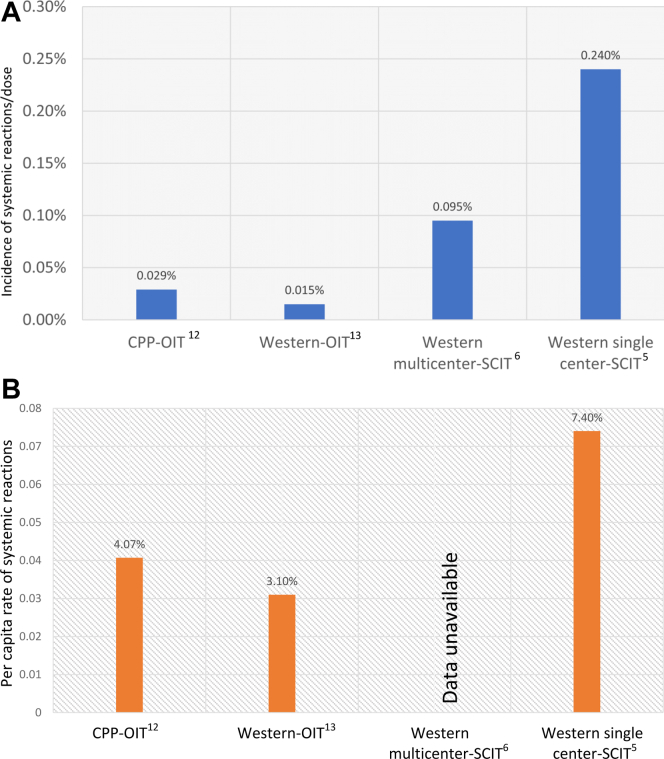
**A,** Incidence of systemic reactions requiring epinephrine in recent Canadian OIT and SCIT studies. **B,** Per capita rate of systemic reactions requiring epinephrine in recent Canadian OIT and SCIT studies.

Comparing these results with those of recent Canadian aeroallergen SCIT studies reveals higher rates of adverse outcomes. A single-center retrospective review of 380 patients from a community allergy and immunology clinic between 2011 and 2017 showed that the incidences of systemic reaction requiring epinephrine were 28 in 380 patients (7.4%) and 1 in 1047 injection visits (0.095%).^5^ No number of patients who had dropped out or discontinued SCIT secondary to side effects during the study time frame was reported. Of the patients who experienced systemic reactions, 26 of 28 (92.9%) received epinephrine during their clinic visit, whereas 2 of 28 reactions (7.1%) occurred outside the 30-minute mandatory observation period, with administration of epinephrine occurring outside the clinic setting.^5^ This is comparable to a review of 860 patients receiving either aeroallergen or venom SCIT from a single academic allergy and immunology center with multiple allergists over a 12-month period in 2019, which showed the incidence of systemic reactions requiring epinephrine was 10 in 4242 injections or 0.24% (all of these reactions resulted from aeroallergen SCIT).^6^ The per capita incidence of epinephrine use for this study is unavailable. Patient dropout secondary to side effects was not reported.^6^ Although the study did not report whether epinephrine was administered in a clinical or home setting, 70% of patients reacted within 30 minutes of the mandatory observation window.^6^ No patient mortality was reported in either of these studies. The results of both Canadian peanut Ped-OIT and SCIT studies are summarized in Fig 1.

Reviewing the data for patients outside of Canada shows high variability in the proportion of severe outcomes that are reported for both Ped-OIT and SCIT.^7^^,^^14^^,^^15^ Results from a recent peanut OIT meta-analysis failed to reveal the number of times epinephrine was given as a proportion of OIT doses (during updosing and maintenance); however, they did report that the incidence of systemic reactions requiring epinephrine was 82 in 1000 patients or 0.082%.^7^ The number of patients who discontinued OIT because of adverse events was 87 in 699 or 0.12%. The results from a meta-analysis pooling data from 15 studies looking at the safety of SCIT among other outcomes showed a relative risk ratio of experiencing a systemic adverse event of 1.15 (95% CI = 0.67, 2.00) versus placebo.^3^ This analysis did not report the rate of systemic reactions requiring epinephrine as a proportion of injections given, nor was the number of patients who dropped out secondary to adverse events reported. Additionally, a review of patients receiving aeroallergen SCIT between 2013 to 2017 in a multicenter outpatient allergy practice in Rochester, New York, found an incidence of systemic reactions of 81 in 86,949 injections or 0.09%; patient dropout rates were not reported.^16^ This is similar to the findings of a review of 54.4 million injection visits between 2008 to 2016 in a multicenter study that included centers in the United States, Canada, and Puerto Rico and found an incidence of 8.7 systemic reactions per 10,000 injections (5.6 grade 1 systemic reactions, 2.7 grade 2 systemic reactions, and 0.35 grade 3 systemic reactions per 10,000 injections).^17^ Between 2012 and 2016, 1 grade 4 systemic reaction occurred in every 160,000 injections.^17^ This study did not report the rate of systemic reactions requiring epinephrine as a proportion of injections given, nor did it report patient dropout rates.^17^

## Discussion

Despite differences in Ped-OIT protocols used and age groups studied, the current real-world Canadian data suggest that severe reactions requiring epinephrine use are no more frequent in patients treated with peanut Ped-OIT than in those treated with aeroallergen SCIT. Comparing outcomes between the 2 Canadian peanut Ped-OIT studies revealed that treatment of preschoolers with a slower, more conservative approach to peanut Ped-OIT dose escalation leads to more favorable safety outcomes.^12^^,^^13^ Additionally, both Canadian peanut Ped-OIT studies show safety outcomes similar to those with SCIT to environmental allergens.

More research is needed to confirm these findings, given the differences in age groups targeted by Ped-OIT and SCIT and differences in the setting of dose administration (with SCIT always provided in a clinical setting and Ped-OIT done partly in a clinical setting and partly in a home setting). These differences in setting may have contributed to the increased epinephrine administration seen in the population of patients with SCIT. A further limitation that may have increased epinephrine administration in the population of patients with SCIT is the use of multiple aeroallergens in the injections, which can increase the risk of reactions. There was also significant variability in the reported dropout rates of patients between Canadian Ped-OIT and SCIT studies, with no dropout rates reported by SCIT studies and 10% to 19% dropout rates reported in Ped-OIT studies.^12^^,^^13^ Further research directly comparing and controlling for variables between SCIT and Ped-OIT is needed—the studies reviewed were not designed to undergo head-to-head comparison between the 2 modalities, nor should this article be interpreted to be a direct head-to-head comparison.

There is also evidence that children with peanut allergy who have accidental exposures resulting in severe reactions are not promptly treated with epinephrine.^18^^,^^19^ Clinically, this raises concern, and how peanut OIT affects the rate of severe reactions in children requiring the use of epinephrine is currently unclear. There is significant variability in the current literature regarding whether patients undergo baseline oral challenge before initiation of food Ped-OIT to confirm eligibility for starting Ped-OIT. Given that patients in high-risk populations will experience anaphylaxis with oral food challenge before Ped-OIT initiation, further research should provide separate representation of this initial episode of anaphylaxis from Ped-OIT safety data, similar to how the data were represented in the 2 Canadian peanut Ped-OIT studies.^12^^,^^13^

Patients with food allergy and their parents have repeatedly identified fear and anxiety around unknowns: how sensitive they are to their food allergen(s), how severe a reaction could be, and how they would manage a reaction if one were to occur.^20^, ^21^, ^22^, ^23^, ^24^, ^25^ This has ultimately led to significant disruption in regular social activities experienced in childhood.^26^ As a result, allergists and families need to be provided with a sense of the comparative risk of severe reactions during OIT relative to that during other common allergen immunotherapy modalities such as SCIT to better inform the process of shared decision making. This is information that can be reassuring for families considering treatment with Ped-OIT or for allergists who may be under the impression that Ped-OIT is riskier than SCIT.

## Conclusion

In conclusion, recent real-world data suggest that the safety of preschool peanut OIT or peanut OIT using a slower buildup schedule is comparable to that of SCIT.
